# Supplementary figures and images for: Digital preparation and osteology of the skull of Lesothosaurus diagnosticus (Ornithischia: Dinosauria)
Source: PeerJ. 2015 Dec 21;3:e1494. doi: 10.7717/peerj.1494 (PMC4690377; doi:10.7717/peerj.1494)

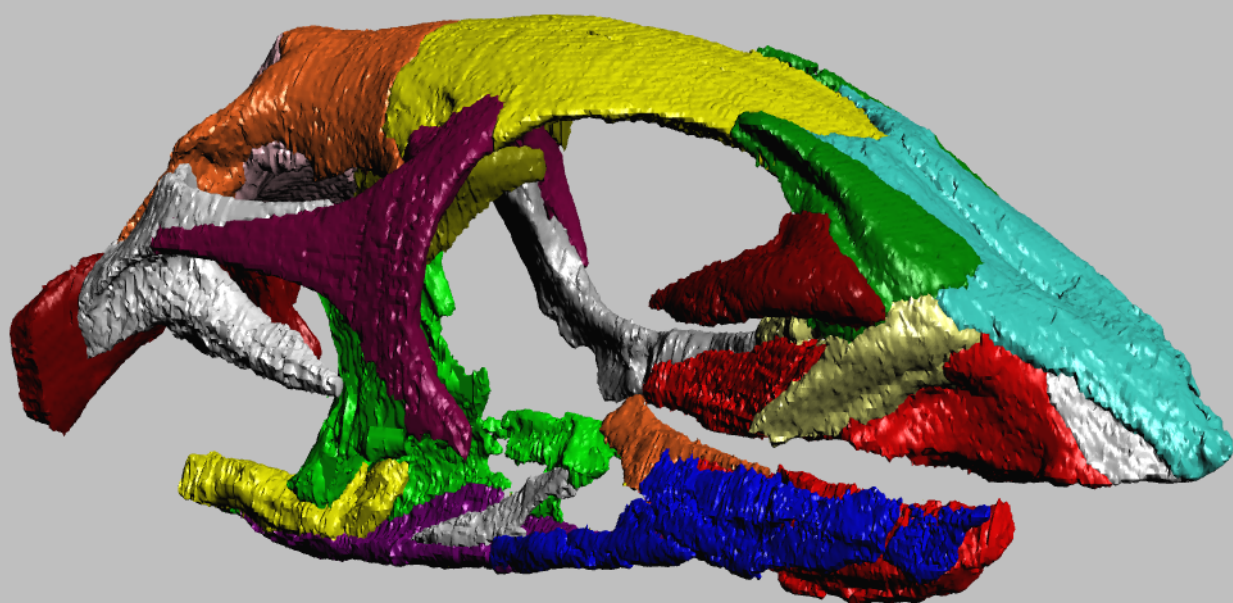

Supplement: Figure S1 [file peerj-03-1494-s001.pdf]

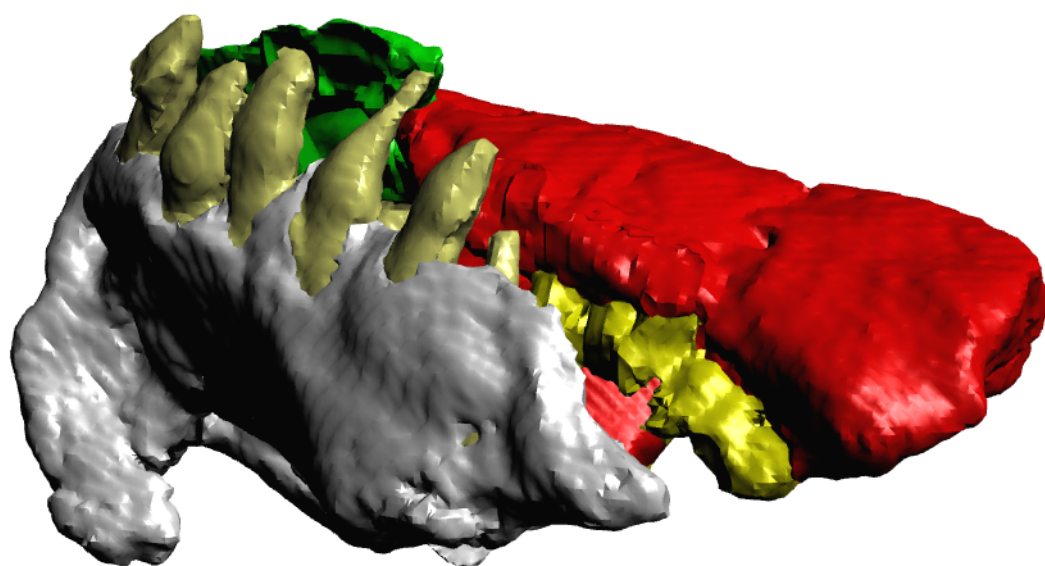

Supplement: Figure S2 [file peerj-03-1494-s002.pdf]

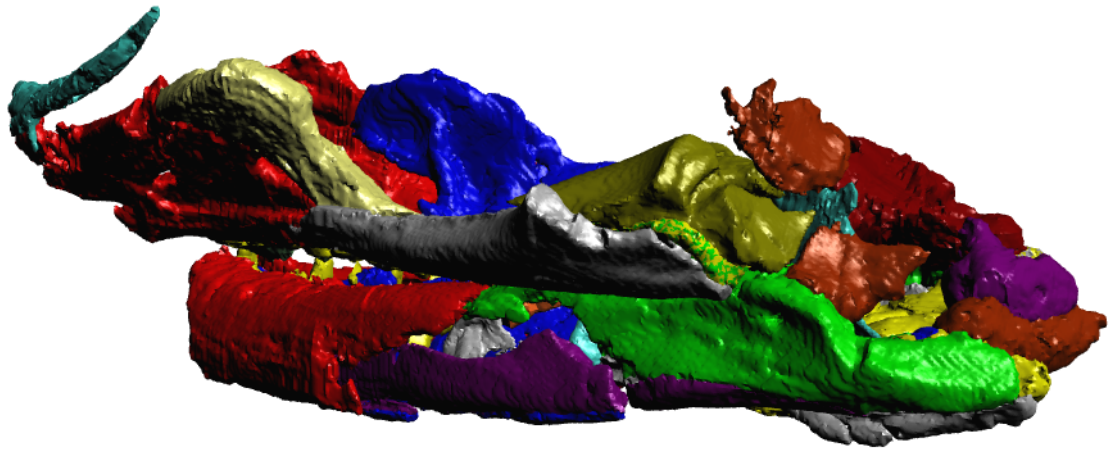

Supplement: Figure S3 [file peerj-03-1494-s003.pdf]

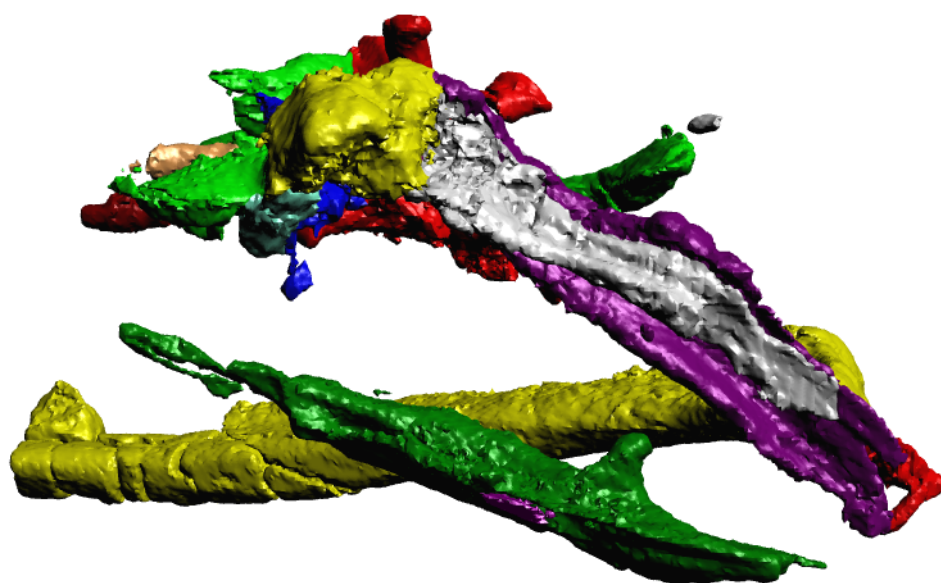

Supplement: Figure S4 [file peerj-03-1494-s004.pdf]
